# *Trichoderma* and Nanotechnology in Sustainable Agriculture: A Review

**DOI:** 10.3389/ffunb.2021.764675

**Published:** 2021-12-01

**Authors:** Claudia A. Ramírez-Valdespino, Erasmo Orrantia-Borunda

**Affiliations:** Laboratorio de Nanotoxicología, Departamento de Medio Ambiente y Energía, Centro de Investigación en Materiales Avanzados, Chihuahua, Mexico

**Keywords:** *Trichoderma*, nanotechnology, metal tolerance, tolerance to NPs, biosynthesis of nanoparticles, mycosynthesis of nanoparticles

## Abstract

Due to their unique properties and functionalities, nanomaterials can be found in different activities as pharmaceutics, cosmetics, medicine, and agriculture, among others. Nowadays, formulations with nano compounds exist to reduce the application of conventional pesticides and fertilizers. Among the most used are nanoparticles (NPs) of copper, zinc, or silver, which are known because of their cytotoxicity, and their accumulation can change the dynamic of microbes present in the soil. In agriculture, *Trichoderma* is widely utilized as a safe biocontrol strategy and to promote plant yield, making it susceptible to be in contact with nanomaterials that can interfere with its viability as well as its biocontrol and plant growth promotion effects. It is well-known that strains of *Trichoderma* can tolerate and uptake heavy metals in their bulk form, but it is poorly understood whether the same occurs with nanomaterials. Interestingly, *Trichoderma* can synthesize NPs that exhibit antimicrobial activities against various organisms of interest, including plant pathogens. In this study, we summarize the main findings regarding *Trichoderma* and nanotechnology, including its use to synthesize NPs and the consequence that these compounds might have in this fungus and its associations. Moreover, based on these findings we discuss whether it is feasible to develop agrochemicals that combine NPs and *Trichoderma* strains to generate more sustainable products or not.

## Introduction

Currently, there is a great demand for food products in the agricultural sector, which means that the soil must contain all necessary nutrients and adequate properties for the optimal development of crops. In addition, crops are susceptible to being attacked by phytopathogens, requiring the use of agrochemicals such as fertilizers and pesticides to counteract the negative effects caused by the pathogen. Recently, the use of agrochemicals containing nanostructured materials has emerged as an alternative in agriculture (Singh et al., 2020).

Among the main nanoparticles (NPs) that have been used in agriculture, standing out are silver NPs (AgNPs), zinc oxide NPs (ZnONPs), and copper oxide NPs (CuONPs), which can be obtained by physical, chemical, and biological methods. Physical and chemical methods, such as ultraviolet radiation, aerosol technologies, lithography, laser ablation, ultrasonic fields, and photochemical reduction techniques, turn out to be expensive methods involving the use and release of toxic compounds during the process, which can cause environmental pollution (Narayanan and Sakthivel, 2010; Guilger-Casagrande and Lima, 2019). On the other hand, the biosynthesis of some types of metal NPs provides a simple, large-scale, economical, and environmentally friendly alternative (Vahabi et al., 2011; Mishra et al., 2014; Saravanakumar et al., 2017; Guilger-Casagrande and Lima, 2019). Moreover, it has been reported that biosynthesized NPs show lower toxicity than NPs obtained by chemical methods due to their stabilization with organic compounds and because no toxic residues are generated during the synthesis process (Fraceto et al., 2018). Furthermore, these new eco-friendly synthesis methods additionally provide better biocompatibility in the use of NPs (Guilger-Casagrande and Lima, 2019).

Biosynthesis can be carried out using plant extracts, bacteria, fungi, algae, yeasts, or bioproducts of their metabolism, including enzymes, agro-industrial residues, and microbial pigments (Gemishev et al., 2019; Elegbede et al., 2020). Similarly, it has been reported that secondary metabolites and other compounds that microorganisms release as a protective mechanism help in metal biosynthesis by converting ions into elemental metal (Vahabi and Dorcheh, 2014; Fraceto et al., 2018).

Thus, molecules released by certain organisms possessing metal resistance can act as reducing and stabilizing agents for the synthesis of metallic NPs (Guilger-Casagrande and Lima, 2019). These stabilizing or protective agents known as cappings, influence the interaction of physicochemical properties of the nanoparticle surface with its environment (Diko et al., 2020). It should be noted that the stability of the cappings depends on the pH: at high pH values, the NPs remain stable in solution, while at low pH values, the proteins that form the cappings denature (Pal and Hossain, 2020).

Filamentous fungi are among the microorganisms with the greatest potential for NP biosynthesis, since their use is more efficient compared with other bioalternatives due to the possibility of easy large-scale cultivation and biomass collection, their high tolerance to metals, resistance to high pressure, and agitation fluxes, as well as the high production of extracellular proteins (Narayanan and Sakthivel, 2010; Vahabi et al., 2011; Salvadori et al., 2014a; Guilger-Casagrande and Lima, 2019). Furthermore, large amounts of extracellular proteins secreted by fungi have been found to increase the synthesis of NPs (Diko et al., 2020). On the other hand, an advantage of using fungi over bacterial systems is that NPs precipitate outside the cell without cellular contaminants and can be directly used in various applications (Narayanan and Sakthivel, 2010). Thus, fungi have been positioned as agents of interest for the biosynthesis of NPs, a process called mycosynthesis. Among the main fungi used are *Fusarium, Aspergillus, Penicillium*, and *Trichoderma* (Bhainsa and D'souza, 2006; Honary et al., 2012; Shelar and Chavan, 2015; Rai et al., 2021).

*Trichoderma* species are widely distributed in the soil and are known to be excellent biocontrol agents and plant growth promoters (Contreras-Cornejo et al., 2009; Hermosa et al., 2012). In addition, these species have started being used in nanotechnology, mainly in the synthesis of metal NPs. More recently, their resistance to different nano compounds has been reported, but little is known about their contribution in the biosynthesis of metallic NPs by their tolerance generated to these compounds and how these affect *Trichoderma* associations (Banik and Luque, 2017; Luo et al., 2018). On the other hand, *Trichoderma* has a high tolerance to metals and it is proposed as a potential bioremediator of contaminated environments (Tripathi et al., 2013). As mentioned above, *Trichoderma* is one of the main fungi used for the mycosynthesis of NPs, suggesting that it may have mechanisms of tolerance to these structures, which could be used synergically to develop products that improve crop weight and treatment against phytopathogens.

**Figure 1:**
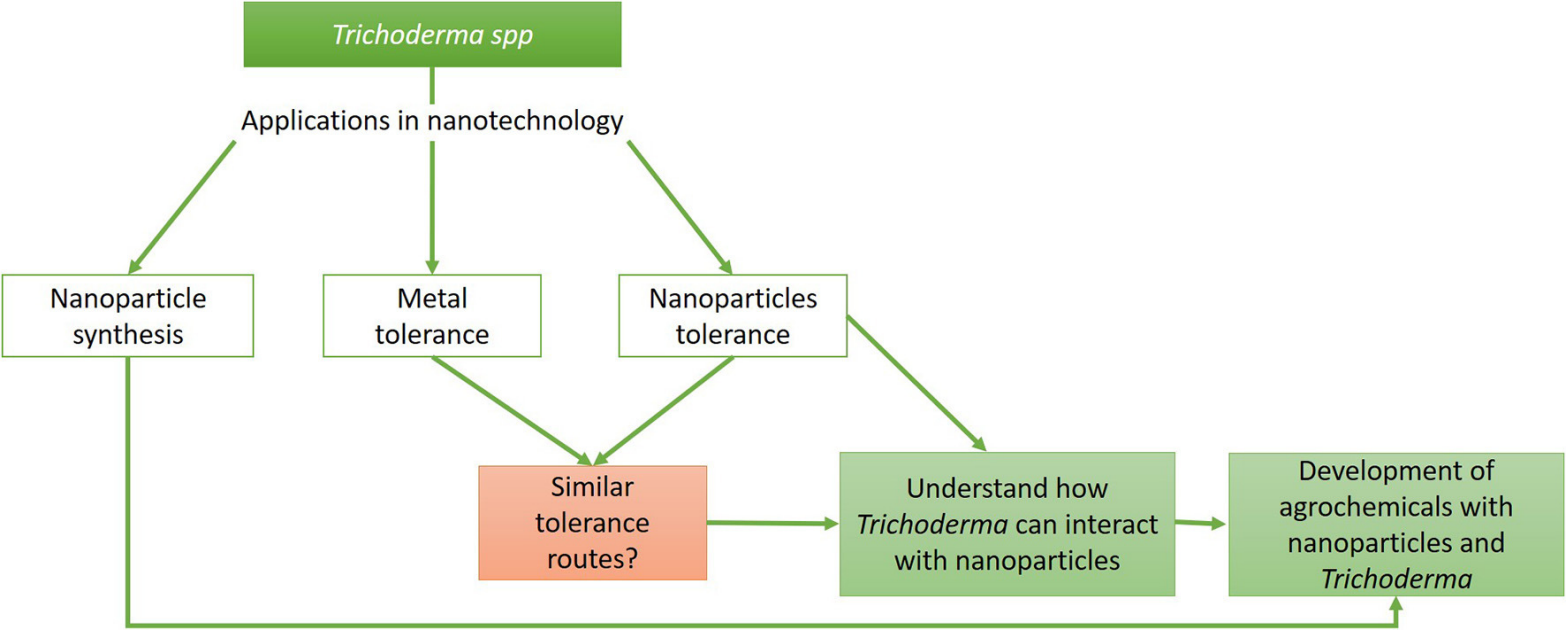
*Trichoderma* and its relevance in nanotechnology. Flow chart representing the different areas where *Trichoderma* can be used in nanotechnology and how these can be united for the generation of products that improve agricultural crops.

Although it is known that the characteristics of bulk material are different from those of an NP, even changing the effects that these structures can have on biological systems, similar tolerance mechanisms can be proposed, helping understand how NPs would be affecting organisms. In this review, we focus on *Trichoderma* and the efforts that the scientific community has made to develop strategies for the biosynthesis of NPs and their effect against microbes, mainly phytopathogens, as well as the mechanisms of tolerance to the metals present in *Trichoderma* and their possible extrapolation to the tolerance that *Trichoderma* shows against NPs. Also, we discuss whether the development of products combining *Trichoderma* and NPs is an option for sustainable agriculture (Figure 1).

## *Trichoderma* as a Synthesizer of NPs

*Trichoderma*-mediated mycosynthesis of NPs can be performed by enzymes such as reductases that can act as bioreductive agents in the biofabrication of NPs (Elegbede et al., 2020). Also, the properties and characteristics of the different metallic NPs obtained suggest that *Trichoderma* is a controllable source of biological synthesis of NPs (Maliszewska et al., 2009; Kareem et al., 2020). In addition, enzymes, proteins, and secondary metabolites that are involved in the biological control of plant pathogens are also generated, which can provide additional biological activity and stability to the obtained NPs (Mishra et al., 2014; Vahabi and Dorcheh, 2014). NPs synthesized by *Trichoderma* spp. have been found to exhibit antimicrobial activity against different microorganisms, especially phytopathogens such as *Fusarium, Aspergillus, Pseudomonas*, and *Xanthomonas* (Ponmurugan, 2017; Consolo et al., 2020; Shobha et al., 2020; Bilesky-José et al., 2021; Boruah and Dutta, 2021).

It is important to consider the parameters used in mycosynthesis and the individual characteristics of the fungal strains to optimize the parameters used to achieve the good mono-dispersity, stability, and biocompatibility of the NPs (Guilger-Casagrande and Lima, 2019). For example, it has been reported that the main factors controlling the nucleation and subsequent formation of metallic NPs, as well as their size and shape, are: fungal growth conditions, reagent concentration, reaction time, initial pH, and temperature (Gemishev et al., 2019). Among the main NPs synthesized by *Trichoderma* are gold (AuNPs), ZnONPs, copper (CuNPs) and CuONPs, selenium (SeNPs), and AgNPs, which show different characteristics and antimicrobial activities (Table 1). Some of the main NPs biosynthesized by *Trichoderma* and their mechanism of mycosynthesis are summarized below.

**Table 1:** Nanoparticles (NPs) synthesized by *Trichoderma*.

**NPs**	**Strain**	**Size (nm)**	**Shape**	**Tested against**	**References**
Ag	*T. asperellum*	4–14 Polydispersed	~Spherical	No tested	Ahmed and Dutta, 2019
	*T. atroviride*	10–15	Spherical	Bacteria, *Candida* sp. and *Aspergillus* sp., *Fusarium* sp.	Abdel-Kareem and Zohri, 2018
		15–25	Anisotropic	*Escherichia coli, Pseudomonas aeruginosa*, and *Staphylococcus aureus*	Saravanakumar et al., 2017
	*T. atroviride, T. atroviride, T. afroharzianum, T. brevicompactum, T. dorothopsis, T. koningiopsis, T. brevicompactum, T. dorothopsis, T. citrinoviride, T. asperellum, T. harzianum, T. brevicompactum, T. atroviride, T. virens, and T. dorothopsis*	5–50	Spherical and oval	*Sclerotinia sclerotiorum*	Tomah et al., 2020
	*T. harzianum*	~50	Spherical and irregular	*S. aureus* and *Klebsiella pneumonia*	Ahluwalia et al., 2014
		5–18	Spherical	*Alternaria alternata, Pyricularia oryzae*, and *S. sclerotiorum*	Consolo et al., 2020
		20–30	Spherical	*S. sclerotiorum*	Guilger et al., 2017
		12.7	Spherical	Not tested	El-Moslamy et al., 2017
		16–63	Spherical and ellipsoidal	Not tested	Shelar and Chavan, 2015
		3–20	Spherical	*Clavibacter michiganensis, Streptococcus thermophiles*, and *Bacillus subtilis*	Noshad et al., 2019
		10–20	Oval	*Aedes aegypti*	Sundaravadivelan and Padmanabhan, 2014
		88–182	Spherical-Rod and Prism	*S. sclerotiorum*	Guilger-Casagrande and Lima, 2019
	*T. interfusant* (FU21)	59.66	Spherical	*Sclerotium rolfsii*	Hirpara and Gajera, 2020
	*T. logibrachiatum*	10	Spherical	*Fusarium verticillioides, F. moniliforme, Penicillium brevicompactum, Helminthosporium oryzae*, and *Pyricylaria grisea*	Elamawi et al., 2018
		61	Spherical, triangular, and cuboid	Halotolerant SRB mixed culture	Omran et al., 2019
	*T. reesei*	15–25	No specified	Not tested	Gemishev et al., 2019
		5–50	Variable	Not tested	Vahabi et al., 2011
	*T. virens VN-11*	8–60	Isotropic	No tested	Devi et al., 2013
	*T. viride*	Not specified. Polydispersed	Nanobowls	*B. subtilis* and *Klebsiella planticola*	Chitra and Annadurai, 2013
		1–50	Globular	*S. aureus, Shigella boydii, Acinetobacter baumanni, Shigella sonnei*, and *S. typhimurium*	Elgorban et al., 2016
		2–4	Spherical	No tested	Fayaz et al., 2010
		100–250	Spherical	*F. moliniforme* and *Rhizoctonia solani*	Saravanakumar and Wang, 2018; Manikandaselvi et al., 2020
	*T. viride, T. hamatum, T. harzianum, and T. koningii*	40–60	Spherical	*F. solani, Fusarium semitectum, Fusarium oxysporum*, and *Fusarium roseum*	El-Wakil, 2020
	*Trichoderma koningii*	8–24	Spherical	*Salmonella typhimurium*	Tripathi et al., 2013
ZnO	*T. harzianum*	134–200	Fan/bouquet structure	*A. alternata, P. oryzae*, and *S. sclerotiorum*	Consolo et al., 2020
	*T. harzianum and T. reesei*	12–35	Hexagonal wuetzite and peaks	*Xanthomonas oryzae pv. Oryzae*	Shobha et al., 2020
Au	*T. hamatumSU136*	5–30	Spherical, pentagonal, and hexagonal	*B. subtilis* ACCB 133, *S. aurous* ACCB 136, *P. aeruginosa* ACCB 156, and *Serratia* sp. ACCB 178	Abdel-Kareem and Zohri, 2018
	*T. harzianum*	30	Spherical	No tested	do Nascimento et al., 2021
		26–34	Spherical	Not tasted	Tripathi et al., 2014
		26–34	Spherical	*E. coli* MTCC 1302	Tripathi et al., 2018
	*T. koningii*	5–40	Small spheres to large polygons (triangles and hexagons)	No tested	Maliszewska et al., 2009
	*T. longibrachiatum*	102.93–123.99	Spherical and oval	*Aspergillus niger, A. flavus, and A. fumigatus; E. coli, Klebsiella granulomatis, P. aeruginosa*, and *S. aureus*	Elegbede et al., 2020
	*T. viride*	59	Spherical	*P. syringae, E. coli, Shigella sonnei*	Mishra et al., 2014
Se	*T. harzianum JF309*	60	Irregular	*Fusarium* sp.*, Alternaria* sp.	Hu et al., 2019
	*T. harzianum, T. atroviride, T. virens, T. longibrechiatum, and T. brevicompactum*	49.5–312.5	Hexagonal, near spherical, and irregular	*Sclerospora graminicola*	Nandini et al., 2017
	*Trichoderma* sp. *WL-Go*	20–220	Spherical and pseudo-spherical	Not tested	Diko et al., 2020
Si and Cu	*T. atroviride*	55.5 (Si), 56 (Cu)	Irregular spherical	*Poria hypolateritia* and *Phomopsis theae*	Natesan et al., 2021
Au and Ag	*T. atroviride*	10–75	Triangular and spherical	*P. theae*	Ponmurugan, 2017
α-Fe_2_O_3_	*T. harzianum*	207	No specified	*S. sclerotiorum*	Bilesky-José et al., 2021
CdS	*T. harzianum*	3–8	Spherical	No tested	Bhadwal et al., 2014
Chitosan	*T. viride*	300	Nearly spherical	*F. oxysporum, R. solani*, and *S. rolfsii*	Boruah and Dutta, 2021
TiO_2_	*T. viride*	74.4	Spherical	*Hecoverpa armigera*	Chinnaperumal et al., 2018
Cu	*T. koningiopsis*	87.5	Spherical	Not tested	Salvadori et al., 2014a
	*T. asperellum*	10–190 nm	Spherical	Not tested	Saravanakumar et al., 2019

*The Trichoderma strain, type, size, and shape of the NPs are shown, as well as the organisms against they have been tested*.

### Biosynthesis of AgNPs

Silver NPs are of great interest due to their high bioactivity and broad antimicrobial spectrum, including plant pathogens (Tomah et al., 2020). Therefore, the use of AgNPs can improve the efficiency of agrochemicals as well as reduce the use of pesticides and biocides (Ahmed and Dutta, 2019).

Two mechanisms are used for the mycosynthesis of NPs: intracellular or extracellular, the extracellular method being the most used because it does not require the extraction of NPs from the cells. After all, only biomolecules secreted by the fungus catalyze a reduction of the metal precursor (AgNO_3_ salt) generating AgNPs (Guilger-Casagrande and Lima, 2019). Moreover, mycosynthesis can be induced to obtain different characteristics of AgNPs that will depend on the fungal culture and production conditions (El-Moslamy et al., 2017).

It has been reported that the main enzymes involved in the biosynthesis of metal NPs in fungi are reductases (Vahabi and Dorcheh, 2014). Among this group of enzymes, nicotinamide adenine dinucleotide (NADH)-dependent nitrate reductase stands out, which is involved in the process of metal ion reduction (Gherbawy et al., 2013; Vahabi and Dorcheh, 2014; Guilger-Casagrande and Lima, 2019). For example, *T. reesei* produces extracellular enzymes that can reduce silver ions to AgNPs, with NADH-dependent nitrate reductase being the agent responsible for carrying out the reduction of Ag^+^ metal ions into metal AgNPs (Gemishev et al., 2019). The reduction occurs due to the transfer of an electron from NADH with nitrate reductase acting as the electron carrier. Proteins can also bind to NPs and improve stability (Pal and Hossain, 2020). Furthermore, Vahabi and Dorcheh (2014) described that the reduction of silver to NPs is also possible by anthraquinones, naphthoquinones, and quinine derivatives, which can act as electron carriers in the reaction.

In the study by Saravanakumar et al. (2017), the authors reported that the size of the NPs varied according to the concentration of AgNO_3_, obtaining dispersed AgNPs, anisotropic in structure, and with an average size of 15–25 nm. Gemishev et al. (2019), biosynthesized AgNPs extracellularly from an AgNO_3_ solution and used filtrates of *T. reesei* grown on different media. It was observed that the optimal medium contained 0.1% corn maceration liquor, 10% extracted biomass, and a concentration of 10 mM AgNO_3_ as a precursor, which produced non-toxic NPs with sizes between 15 and 25 nm in the crystalline phase, narrow size distribution, and improved stability among particles. On the other hand, in the study of Noshad et al. (2019), who applied an extract of *Trichoderma harzianum* to two different concentrations (1 and 2.5 mM) of AgNO_3_, AgNPs with sizes between 3 and 20 nm were generated. Furthermore, it was observed that the 1 mM concentration produced AgNPs with a smaller size and better antimicrobial efficacy.

Sundaravadivelan and Padmanabhan (2014) obtained oval and monodisperse AgNPs with sizes between 10 and 20 nm by using the same concentration of AgNO_3_ and incubating for 24 h. Interestingly, Shelar and Chavan (2015) worked with a 1 mM concentration of AgNO_3_ and *T. harzianum* and obtained polydisperse, spherical, and ellipsoidal NPs with a size range of 19–63 nm at 3 h of exposure. The difference in the NP sizes reported in these two studies can probably be attributed to the variation in the exposure times to AgNO_3_ concentration.

Ramos et al. (2020), applied pH and reaction time variations for biosynthesis optimization using *Trichoderma* isolate TCH 01 and found that pH 5, 9 days of exposure, and 1 mM AgNO_3_ resulted in smaller sized NPs (150 nm) and better polydispersion rates. Similarly, Pal and Hossain (2020) sought to optimize the pH, substrate concentration, and incubation period to obtain a better quality and quantity of AgNPs. Their results suggested that pH 9, 72 h incubation period, and 2 mM AgNO_3_ were the optimal conditions to obtain a spherical, monodisperse, and stable form of AgNPs. The optimal conditions discussed by the authors may diverge due to the existence of a dependence of the factors (pH, incubation time, and AgNO_3_ concentration).

Regarding the optimal parameter settings for the mycosynthesis system, El-Moslamy et al. (2017), applied the Taguchi design using a *T. harzianum* strain. They succeeded in increasing the biomass yield in the production of AgNPs in the medium. The optimal conditions observed were as follows: a concentration of AgNO_3_ at 0.01 M, diluted reductant (10v/v, pH 5), and incubation at 30°C, 200 rpm for 24 h.

Research in this regard has allowed the understanding of some important mechanisms that take place in mycosynthesis, which enables the optimization of the parameters used to generate specific characteristics in AgNPs, such as dispersion, stability, and biocompatibility. Altogether, these previous studies suggest that the characteristics of the obtained NPs depend on the fungi culture and the physicochemical conditions of production.

### Biosynthesis of SeNPs

Selenium (Se) has beneficial effects at low concentrations on plant and animal metabolism, at the same time, it is involved in the protection against reactive oxygen species (ROS) in the form of selenoproteins (Bărbieru et al., 2019). Thus, SeNPs can be an alternative to the use of Se salts.

Regarding the synthesis of SeNPs, Hu et al. (2019) performed a comparison between a traditional synthesis (SNP, SeNPs) and biosynthesis (TSNP, SeNPs by *Trichoderma*) method of SeNPs. For biosynthesis, a concentration of 5 mM NaSeO_3_ was applied to a solution of metabolites of eight *Trichoderma* species. The results obtained with the SNP method were: SeNPs with spherical and pseudospherical shapes of 50 nm, as well as the presence of polysaccharides. As for the SeNPs obtained by the TSNP method, they reported a size of 60 nm with an irregular shape, probably caused by the capping effect. At the same time, the study revealed an increase in the presence of amide materials in TSNP compared with SNP, which act as stabilizing agents (cappings). On the other hand, other studies such as that by Nandini et al. (2017), evaluated the production of SeNPs using three different media: culture filtrate (CF), cell lysate (CL), and crude cell wall (CW). They used six different species of *Trichoderma* spp. in each medium and applied a concentration of 25 mM Na_2_SeO_3_, obtaining SeNPs from 49.5 to 312.5 nm in all media. Based on the results, the best medium for NPs production was CF, as it facilitated the process compared with CL and CW. Other elements were also analyzed in mycosynthesis by Diko et al. (2020), who studied the effects of pH concerning inoculation time and SeO_2_ concentration. The optimal conditions (pH of 8, 2 mM SeO_2_ concentration, 24 h of SeO_2_ inoculation, and *Trichoderma* sp.) produced spherical and pseudospherical SeNPs of 20–220 nm with the presence of alkene, alkane, and alcohol functional groups, thus suggesting their participation in the SeNPs synthesis reaction as cappings.

In contrast to the numerous studies on AgNPs, there is still little research on the synthesis of SeNPs. However, it can be suggested that SeNPs synthesized by *Trichoderma* have potential as an alternative to the use of Se salts and SeNPs originating from physical and chemical methods, since they present antioxidant and catalytic properties and can be used in biomedical, electronic, optical, and chemical fields.

### Biosynthesis of AuNPs

Gold NPs can be used as catalysts, antimicrobial agents, or in the biomedical field as diagnostics, biomolecule detection, and nano-drug carriers (do Nascimento et al., 2021). For example, several studies have reported that AuNPs have antimicrobial activity against pathogens such as *Aspergillus niger, Aspergillus flavus, Aspergillus fumigatus, Escherichia coli, Klebsiella granulomatis, Pseudomonas aeruginosa, Staphylococcus aureus*, and *Shigella sonnei* (Mishra et al., 2014; Abdel-Kareem and Zohri, 2018; Tripathi et al., 2018; Elegbede et al., 2020). Moreover, the biosynthesis of AuNPs is presented as a low-cost and eco-friendly alternative for gold recovery, compared to electrochemical treatments of reverse osmosis and ion exchange resins (do Nascimento et al., 2021). In the study conducted by do do Nascimento et al. (2021), it was shown that *T. harzianum* has a gold biosorption capacity of ~1,340 mg of the metal per g of biomass in 180 min, with a mycosynthesis of spherical NPs with a size below 30 nm. Regarding the study of the optimal parameters for mycosynthesis, Abdel-Kareem and Zohri (2018) exposed *T. hamatum* SU136 to three different concentrations of Au_2_Cl_6_ (0.25, 0.5, and 1 mM). All conditions generated AuNPs, but the optimal parameters for obtaining smaller NPs were: 0.5 mM Au_2_Cl_6_ at pH of 7 and 38°C. Similarly, Maliszewska et al. (2009) took concentration as the main variable, using the HAuCl_4_ solution. Their results suggested that the presence of free and exposed thiol (-SH) groups are essential for the reduction of gold ions. In the mycosynthesis performed by Tripathi et al. (2014), the authors contacted 5 g of wet biomass with 1 mM HAuCl_4_ during a 72 h incubation period. The results showed a complete reduction of Au^3+^ into Au^0^ generating spherical NPs of 26–34 nm. They observed that under metallic stress conditions *T. harzianum* synthesized cysteine extracellularly, which encapsulates the AuNPs to reduce the toxic effect of gold ions while stabilizing the NPs. Mishra et al. (2014), analyzed the effects of various other parameters such as temperature, pH, cell extract concentration, and HAuCl_4_ concentration. The results showed that NPs were obtained at pH 7 and 9 but not at a pH of 5; whereas for the temperatures analyzed (20, 30, 40, 40, 50, and 100°C), it was found that the NPs are smaller than 20 nm in size at 100°C; concerning the cell extract concentrations of *Trichoderma viride* cell extract concentrations studied (100, 50, and 10%), NPs were synthesized in less time at 10%; finally, for the HAuCl_4_ concentrations (500 and 250 mg/L), NPs were obtained after 10 min only at 250 mg/L.

These studies indicate that the biosynthesis of AuNPs mediated by *Trichoderma* allows obtaining AuNPs with unique optical, thermal, chemical, and physical properties, which can also be used as a method for gold recovery.

### Biosynthesis of CuNPs and CuONPs

Copper NPs have a wide number of applications, these can be used as high-temperature conductors, gas sensors, catalysts, in solar cells, and for wood treatment (Salvadori et al., 2014b). It has also been reported to exhibit antimicrobial activity against phytopathogens (Natesan et al., 2021). Their antimicrobial activity can be enhanced by their easy interaction with other particles, which is caused by the high surface-to-volume ratio (Al-Hakkani, 2020).

The amino and aromatic groups of secondary metabolites have been reported to act as encapsulants or reducing agents in the formation of CuONPs by *T. asperellum* (Saravanakumar et al., 2019). In the mycosynthesis of CuNPs performed by Salvadori et al. (2014b), they used the dead biomass of *T. koningiopsis*, because it presents a higher affinity to copper and adsorption capacity for metal ions (21.1 mg/g) when compared with live biomass and other biosorbents. The results obtained showed that 90% of the NPs were obtained in 60 min, which was considered a fast process. The predominant shape of the CuNPs obtained by the authors was spherical with an average diameter of 87.5 nm.

Little is known about this topic and it is necessary to generate a greater number of studies on the mycosynthesis of Cu and CuONPs for a better understanding of the mechanisms of action of the production, considering the potential application that these NPs present.

### Biosynthesis of ZnONPs

Among the NPs that have been most widely used in agriculture, ZnONPs stand out as beneficial to plants and the soil microbiota (Shobha et al., 2020). Similarly, they possess antifungal activities against *Fusarium* spp., *Botrytis cinerea, Penicillium expansum, A. niger*, and *Rhizhopus stolonifera*. Similarly, ZnONPs are also considered effective antibacterial agents against a broad spectrum of species (Consolo et al., 2020). For example, Shobha et al. (2020) synthesized ZnONPs using three different *Trichoderna* isolates and tested their action against *Xanthomonas oryza*e pv. Oryzae. The ZnONPs they obtained presented unique shapes (hexagons and peaks) in the range of 12–35 nm, which could be easily and sustainably produced on a large scale.

Previous research indicates that ZnONPs can be used as antimicrobials and as an alternative to chemical fertilizers in agriculture, being an environmentally friendly method of large-scale production. However, more research is needed to fully decipher the best conditions to obtain NPs with the desired characteristics and to obtain better results when used in different areas, including agronanotechnology.

## Effect of NPs in *Trichoderma*

The anthropogenic use of products containing different types of NPs is increasing, suggesting their imminent arrival in different ecosystems, including aquifers or agricultural soils. Some reports indicate that the presence of NPs can induce changes in microbial communities, even affecting the beneficial associations that these microorganisms can form with plants (Ameen et al., 2021). For example, Bandyopadhyay et al. (2012) analyzed the effect of ZnONPs on the nitrogen-fixing bacterium *Sinorhizobium meliloti*, a symbiotic bacterium associated with alfalfa, finding that they were highly toxic. Another study indicated that FeONPs at 3.2 mg/kg significantly reduced the mycorrhizal clover biomass by 34% by reducing the nutrient acquisition by the arbuscular mycorrhiza fungi (AMF) roots. In contrast, with AgNPs, no negative effects were observed at concentrations above 0.1 mg/kg; however, AgNPs at 0.01 mg/kg inhibited the growth of mycorrhizal clover (Feng et al., 2013). Cao et al. (2017), also worked with AgNPs and showed that there is an inhibition of the interaction of maize plants with arbuscular mycorrhizae, causing a significant decrease in root mycorrhizal colonization rate, soil alkaline phosphatase activity, available phosphorus (P) content, and plant P nutrition. These studies suggest that the effect that NPs will have on beneficial organisms and their associations with plants are highly varied, depending on the type of NPs, concentrations, and participating organisms.

**Figure 2:**
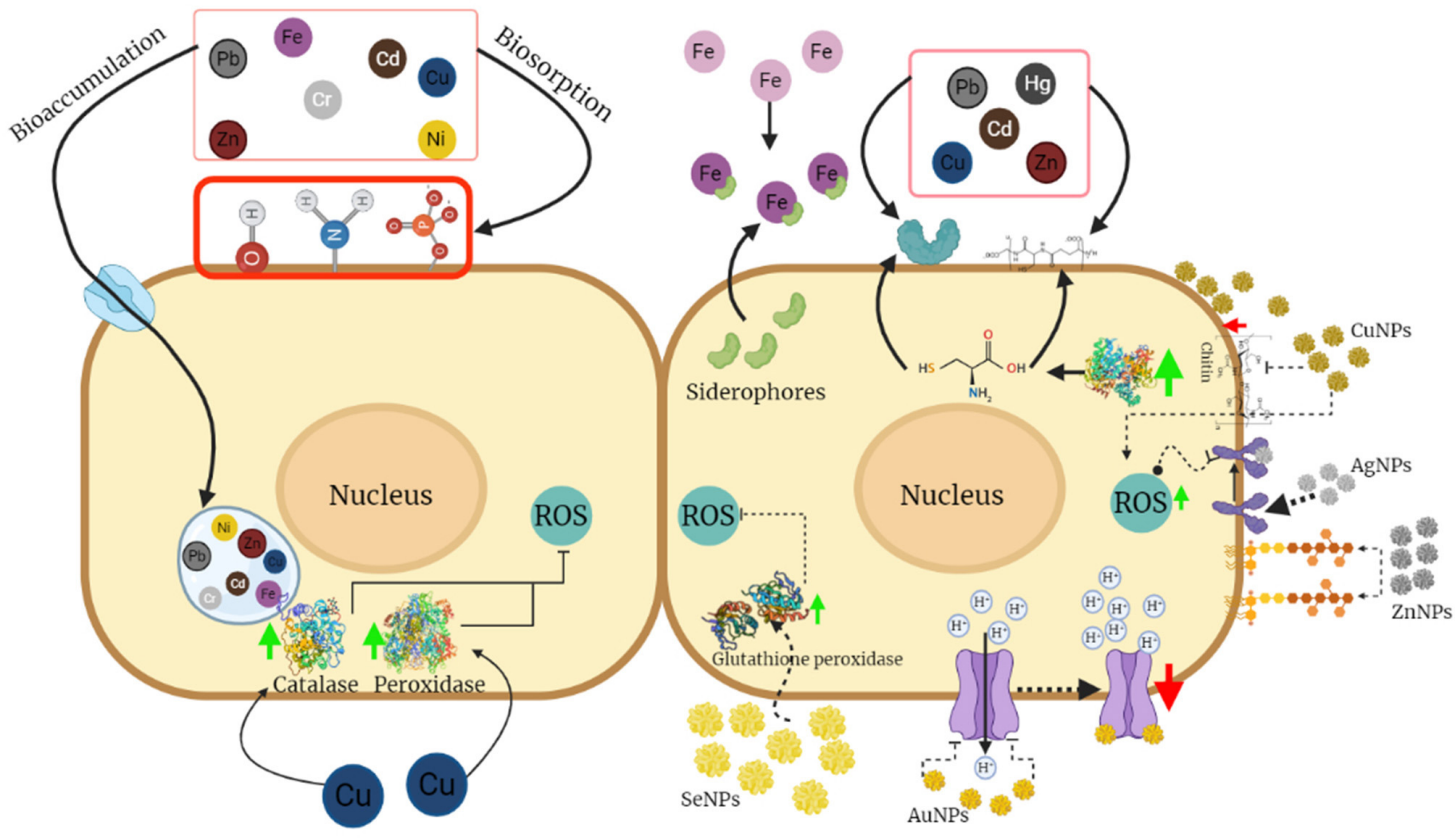
Mechanisms of tolerance to metals and nanoparticles (NPs) in *Trichoderma*. Most of the mechanisms of tolerance to metals are based on processes of adsorption and absorption in the cell walls due to the presence of functional groups, proteins, or compounds that serve as chelating agents, as well as the accumulation of these in the vacuoles. Mechanisms involving the activity of antioxidant enzymes that reduce the damage caused by reactive oxygen species might also be present. This figure was created by BioRender.com.

So far, the study of the effect of NPs on fungi biocontrol is relatively new and there is limited information about it; however, studies on the effect of NPs on phytopathogenic fungi, such as *Aspergillus, Fusarium*, and *Rhizopus*, can provide information about their resistance mechanisms to propose possible strategies that can be presented in *Trichoderma* species giving them the advantage to tolerate higher concentrations of NPs than phytopathogens (Figure 2). The effects of different metal NPs on fungi that have been described are described below.

### Effect of SeNPs

Selenium is a micronutrient that is incorporated into the structure of enzymes such as glutathione peroxidase, iodothyronine disiodase, and thioredoxin reductase, involved in the antioxidant response, detoxification processes, and cell growth, respectively (Forootanfar et al., 2014; Shakibaie et al., 2015). SeNPs have biomedical applications, such as treatments for cancer, diabetes, or inflammatory disorders, and have antioxidant, antimicrobial, and antiviral properties (Chen et al., 1992; Tseng, 2004; Messarah et al., 2012; Khurana et al., 2019).

Its antifungal effect was evaluated in *A. niger*, where at concentrations between 250 and 1,000 μg/ml diminished fungal growth; conversely, at subinhibitory concentrations (10 μg/ml) *A. niger* growth was stimulated, possibly since being a micronutrient, small concentrations of NPs can be used as cofactors by the enzymes mentioned above (Kazempour et al., 2013). In *T. virens*, the secretion of a thioredoxin reductase was reported during its interaction with maize plants (Nogueira-Lopez et al., 2018), suggesting that SeNPs could have an effect on the associations that *Trichoderma* has with plants. However, it is indispensable to further analyze whether this effect is positive or negative.

### Effect of AuNPs

Gold NPs possess different physical and chemical properties that make them excellent structures for the fabrication of novel chemical and biological sensors. Moreover, they have applicability in different areas due to their optoelectronic properties, high biocompatibility, and antimicrobial activity (Das et al., 2011).

Studies have shown that AuNPs have an antifungal effect against *A. niger* and *A. flavus*, even better than commercial antifungal drugs (Jayaseelan et al., 2013). Interestingly, it is known that the antifungal effect of these NPs is enhanced when applied in conjunction with other materials such as chitosan, in addition to other factors such as the dose and concentration of the NPs. For example, the combination of these two nanomaterials is able to inhibit up to 100% of the growth of *F. oxysporum* when treated with doses up to 5 ml with 25, 50, or 75 μg/ml. These effects may be due to the fact that AuNPs have the ability to interact with enzymes involved in the regulation of the proton gradient to the extracellular medium, causing their inactivation, which would result in cell death (Lipşa et al., 2020).

In *Trichoderma*, its proton transport systems are efficient when facing different types of stress, managing to regulate pH, and maintaining optimal growth (Ali and Hashem, 2007). Therefore, the mechanism of tolerance that *Trichoderma* could present against AuNPs would be a positive regulation in the synthesis of proton transporters, decreasing the imbalance of protons and leading to greater resistance to these compounds compared with phytopathogenic fungi.

### Effect of ZnONPs

Zinc oxide NPs have been used as an antimicrobial agent due to their effectiveness, low cost, low toxicity to human cells, and stability (Sirelkhatim et al., 2015). In addition to that, their antifungal effects have been demonstrated against phytopathogenic fungi such as *Fusarium graminearum* and the biocontrol fungus *T. viride*. In the case of *F. graminearum*, there is evidence that the size of NPs is an important factor in the antifungal activity; for example, in a study by Dimkpa et al. (2013) where the antifungal effect of ZnONPs was compared with the zinc oxide micro-particles on *F. graminearum*, it was shown that ZnONPs exhibit a greater inhibition in fungal growth as opposed to zinc oxide micro-particles, this was due to the fact that NPs present a greater surface area compared with micro-particles, which causes a greater interaction with the fungus. On the other hand, studies on *T. viride* show that the presence or absence of light is another factor to consider when working with ZnONPs. Luo and coworkers (2018), observed a higher percentage of inhibition by ZnONPs in the presence of light, reaching up to 99.26% compared with 8.15% shown in darkness. Similarly, it is shown that the capping of NPs presents an increase in the inhibition efficiency, mainly because capping reduces the aggregation of NPs by increasing the surface area, which generates a greater contact surface with compounds in the fungal cell wall, such as lipopolysaccharides, and exerting more intense stress than that generated by particles or zinc salts that have much larger sizes.

### Effect of AgNPs

Due to their physicochemical and biological properties, AgNPs are widely used in various fields, such as sensors, antimicrobial agents, and catalysis (Zhang et al., 2014; Baghayeri et al., 2016; Saha et al., 2017).

Studies have shown that the use of AgNPs as antifungals reduces even by 90%, the growth of some plant pathogens, such as *Fusarium verticillioides, Fusarium moniliforme, Penicillium brevicompactum, Helminthosporium oryzae*, and *Pyricularia grisea* (Elamawi et al., 2018). In *Rhizopus* sp. and *Aspergillus* sp., the AgNPs inhibited the hyphal growth, in addition to the detrimental effects on conidia germination and other deformations in the cell membrane structure and the inhibition of the normal development of both strains was observed (Medda et al., 2015).

The effect of these NPs is possibly due to the high affinity of AgNPs to interact and bind to external membrane proteins, affecting their integrity, forming ROS, and causing cell death (Ouda, 2014; Mahdizadeh et al., 2015; Medda et al., 2015; Elamawi et al., 2018). Another study reported that AgNPs almost completely inhibited the growth of *Pythium aphanidermatum* and *Sclerotinia sclerotiurum*, while *T. harzianum* had an inhibition percentage of about 80% (Mahdizadeh et al., 2015). A possible mechanism explaining the higher tolerance of *Trichoderma* to AgNPs could be its ability to neutralize ROS by increasing the activity of antioxidant enzymes such as catalase and peroxidase, which have been shown to increase considerably in the presence of this type of stress, which could open the possibility of using these NPs in the control of phytopathogenic species without compromising the biocontrol fungal species.

### Effect of CuNPs and CuONPs

Copper NPs have gained special attention in areas such as the agricultural industry because they have high selectivity against microorganisms at low concentrations, low toxicity to humans, and their production costs are lower compared with the synthesis of Ag and AuNPs (Pham et al., 2019; Maqsood et al., 2020).

The antifungal activity of CuNPs has been studied on *Fusarium* and *Aspergillus*, and it has been shown that the efficiency of the inhibition of fungal growth depends on factors such as concentration and NP size. Thus, when concentration is taken into account, *Fusarium* and *Aspergillus* growth inhibition percentages of up to 93.98 and 97% have been obtained at concentrations of 450 and 500 ppm, respectively (Viet et al., 2016; Pariona et al., 2019). On the other hand, when the size of the NPs is considered, it is possible to obtain a high inhibition percentage with much lower concentrations, as is shown in the study of Pham et al. (2019) who obtained an inhibition percentage of 72% at a concentration of 20 ppm when the NPs are 53 nm in average size.

One of the reasons why fungal growth is inhibited by CuNPs is because they cause changes in morphology, ranging from deformations, rugosities, and weakening in the mycelium, or even non-germinated conidia, indicating a deleterious effect on the cell wall that leads to the expulsion of intracellular components and ultimately, cell death. These effects might possibly be due to disruption of the synthesis of cell wall compounds, such as chitin, causing a loss of cell wall stability and generating deformations and osmotic imbalances, thus facilitating the permeation of CuNPs (Pariona et al., 2019).

Banik and Luque (2017) showed that CuONPs did not affect the growth of *T. harzianum* at the presence of 200 mg/mL and only an effect in conidiation was observed. These results contrast with those observed in *Phytophthora cinnamomi* and *Alternaria alternata*, where the presence of 100 and 200 mg/ml of CuONPs, respectively, decreased the fungal growth rate.

It is well known that *Trichoderma* presents a defense response against ROS generated by the presence of metals or other NPs in the extracellular medium. Therefore, as with AgNPs, the increase in the enzymatic activity of catalase and peroxidase, responsible for neutralizing ROS, would be a mechanism of tolerance to CuNPs. Furthermore, considering how copper tolerance is conferred, another possibility is that the NPs accumulate in the *Trichoderma* cell wall, in the same way as it works with bulk copper. Therefore, if *Trichoderma* is able to tolerate higher concentrations of CuNPs compared with phytopathogenic fungi, it would be possible to apply it in the development of CuNPs products to minimize fungal diseases that might harm beneficial species.

## Metal Tolerance of *Trichoderma*

As mentioned above, there are few studies on the tolerance of *Trichoderma* to NPs, and trying to understand how *Trichoderma* can tolerate NPs, an analysis of metal tolerance may serve as a good approach. Below, we review the tolerance mechanisms that *Trichoderma* exhibits against metals (Table 2).

**Table 2:** Mechanisms of tolerance to metals in *Trichoderma*.

**Strain**	**Metal resistance**	**Mechanism**	**References**
*T. asperellum*	Fe, Cu, Mn, and Zn	Production of iron chelators (siderophores)	de Santiago et al., 2011
	Cu	Increase in catalase and peroxidase activity	Juniors et al., 2020
*T. atroviride*	Cu, Cd, and Zn	Biosorption by physical binding to negatively charged groups on the cell wall	Errasquin and Vazquez, 2003
	Zn, Ba, and Fe	Biosorption by functional groups with metal complexion capacity in the cell wall	Kacprzak and Malina, 2005
	Cu	Adsorption into the cell wall and absorption	Yazdani et al., 2010a
*T. brevicompactum*	Cu, Cr, Cd, Zn, and Pb	Biosorption and bioaccumulation by physical binding to negatively charged groups on the cell wall	Zhang et al., 2020
*T. harzianum*	Zn, Pb, Cd, and Hg	Increase of OASTL activity and transformation of cysteine into chelators like metallothioneins, phytochelatins, etc.	Raspanti et al., 2009
	Hg	Synthesis of hydrophobin by up-regulation of the gene encoding hydrophobin	Puglisi et al., 2012
*T. viride*	Zn(II), Pb(II), and Cd(II)	Ion exchange and adsorption	Ali and Hashem, 2007
*Trichoderma* sp.	Ni, Cd, and Cr	Adsorption and absorption by functional groups	Chew et al., 2012
	Pb, Zn, Cd, and Cu	Intracellular accumulation and melanin development	Ayad et al., 2018
*T. asperelloides, T. hamatum*, and *T. harzianum*	Cd, Cu, Hg, and Zn	Production of chelating substances and proteins; metal transport pathways inside the cells	Maldaner et al., 2020
*T. asperellum* and *T. harzianum*	Cd, Pb, and Ni	Biosorption by constituents and functional groups on the cell wall	Hoseinzadeh et al., 2017

*The strain of Trichoderma, the metal(s) to which it is tolerant, and the proposed mechanisms of tolerance are shown*.

Studies on the tolerance and removal capacity of *Trichoderma* have been carried out under different conditions of pH, temperature, and metal concentrations, with the aim of knowing the effects of changing these parameters in the characteristics of the NPs obtained and thus, how they can be used in different environments (Krantz-Rülcker et al., 1993; Ali and Hashem, 2007).

Several authors have suggested that the mechanisms responsible for metal tolerance are based on biosorption, as the metal is immobilized on the cell surface (adsorption) or accumulates inside the cell (absorption) (Errasquin and Vazquez, 2003; Kacprzak and Malina, 2005; Yazdani et al., 2010b; Chew et al., 2012; Hoseinzadeh et al., 2017; Zhang et al., 2020). However, the specific mechanism will depend on both the metal and fungal species under study (Errasquin and Vazquez, 2003; Ali and Hashem, 2007). In this section, the mechanisms of tolerance of *Trichoderma* to metals to which it can be commonly exposed in its natural habitat are presented (Figure 2).

### Tolerance to Lead

Among the mechanisms that *Trichoderma* employs when is exposed to lead, it has been found that at high concentrations (up to 4,000 mg/L), the fungus is capable of producing melanin in the chlamydospores to synthesize enzymes that allow it to immobilize lead through its transformation into less toxic species (Povedano-Priego et al., 2017). Similarly, it has been observed that when the pH of the medium changes, ion exchange takes place on the cell surface to maintain an equilibrium in the concentration of protons in the medium. In most cases, this process helps the pH value to approach 6, where the highest removal efficiency is obtained (Ali and Hashem, 2007). These studies suggest that *Trichoderma* can be an excellent candidate for the removal of lead in the soil, based on the great quantities that it can tolerate and the fact that the pH present in agricultural soils is usually slightly acid.

### Tolerance to Cadmium

Although cadmium is an uncommon element and has no known biological function, when introduced into the environment as waste, it can become one of the most toxic metals. In *T. viride*, the fungus can decrease its growth up to 97.2% under concentrations above 125 mg/L of cadmium (Errasquin and Vazquez, 2003; Zhang et al., 2020). On the other hand, it has been demonstrated that pH is a key factor in the tolerance to this metal since a better removal is observed at pH 4, suggesting that a pH below neutrality supposes a positive effect in the tolerance to *Trichoderma*, similar to the tolerance to lead. This effect may be due to the fact that at slightly acidic pH values, the balance between the charges on the cell surface allows a better biosorption of metal ions to the binding sites.

The tolerance to this metal has also been studied from a metabolic point of view to know which processes are induced or repressed by this stress. It has been observed that cysteine metabolism may be involved in cadmium tolerance. For example, an increase in the enzymatic activity of O-acetyl-serine (thiol) lyase (OASTL) (enzyme responsible for the generation of cysteine from sulfur and O-acetyl-serine) occurs when *Trichoderma* is subjected to cadmium stress; in addition, a slight decrease in the cysteine concentration can also be observed, suggesting a relation between the cysteine metabolism and metal tolerance response in *Trichoderma* (Raspanti et al., 2009).

### Tolerance to Zinc

Although zinc is an essential element for organisms, when it is present at high concentrations, it can become toxic. The tolerance to high concentrations of zinc by *Trichoderma* seems to be associated with the availability of carbon sources, it has been demonstrated that in a medium with saline solution, *T. atroviride* is able to remove up to 16 times more zinc than in a nutritive medium. Based on this, two mechanisms of response have been elucidated: the first is a process of active detoxification in the presence of a carbon source; the second is autolysis in the absence of the carbon source. The latter is the most efficient in removal because when the cell is ruptured, a larger surface area of the cell wall is exposed, thus increasing biosorption (Errasquin and Vazquez, 2003). This suggests that *Trichoderma* can be used under limiting concentrations of carbon sources to remove zinc.

### Tolerance to Copper

Copper is the most common pollutant produced as waste from mining, metallurgy, fertilizer use, and the agro-industry, and is also a cofactor of several enzymes involved in different metabolic processes (Yazdani et al., 2010a). However, its excess within cells can cause the generation of ROS (Yazdani et al., 2009; Ayad et al., 2018).

As copper is a common contaminant, its concentrations in soils and water bodies are usually high and the tolerance of *Trichoderma* to this metal is good compared with that shown by other organisms. Tolerance up to 300 mg/L has been observed in both solid and liquid media, demonstrating that *Trichoderma* might be able to be used in bioremediation processes in the soil and water (Errasquin and Vazquez, 2003; Yazdani et al., 2010a; Yap et al., 2011; Maldaner et al., 2020). Anand et al. (2006) reported that *T. viride* is capable to tolerate copper up to a concentration of 200 mg/L, with a limit at 300 mg/L of Cu(II) in which there was no growth. Moreover, *T. viride* bioaccumulates copper as a layer on the cell wall surface as well. Another mechanism involved in copper tolerance showed that enzymes such as catalase and peroxidase increase their activity in the presence of copper, while the malondialdehyde (MDA) levels decrease (Juniors et al., 2020).

Therefore, it can be suggested that the tolerance that *Trichoderma* exhibits to copper are due to biosorption mechanisms where, on the one hand, copper can be absorbed on the cell surface by components that contain negatively charged groups in order to have an affinity for metal ions, or on the other hand, there can be a presence of copper transporting proteins that internalize it into the cell so that it can be used in metabolic processes or accumulated in the vacuole. The latter possibility would imply a highly strict transport regulation since high concentrations of copper inside the cell could cause cell death. In the case of the enzymatic activity of catalase and peroxidase, this could be considered a secondary tolerance mechanism that could act when the biosorption process is not sufficient. These studies suggest that *Trichoderma* has several mechanisms to efficiently tolerate the presence of copper, which represents an advantage in agriculture since several agrochemicals contain this metal in their formulation.

### Tolerance of Chromium

Similar to cadmium, chromium is an extremely toxic element and, like copper, comes from metallurgical processes or the manufacture of batteries or wood preservation processes (Tumolo et al., 2020). Despite its high toxicity to biological systems, it has been found that *Trichoderma* species extracted from groundwater, have a great capacity to tolerate and remove this metal, showing removal efficiencies of up to 83.3% (Chew et al., 2012). However, another study showed removal percentages of just 31.83% when using the species *T. brevicompactum* extracted from the earthworm intestine (Zhang et al., 2020). A possible explanation is that tolerance to these metals may vary according to the environment from which *Trichoderma* is isolated. For example, for species from groundwater, their environment may represent higher stress due to interactions with metals or toxic pollutants, compared with the strain obtained from earthworm gut.

These studies suggest that *Trichoderma* can tolerate large amounts of various metals that, due to anthropogenic activity, can be found in the soil and represent a danger to organisms present in this environment, including crops of agricultural importance. Moreover, this tolerance could be extrapolated to NPs that could be found in the environment where *Trichoderma* is present.

## Conclusions

The presence of *Trichoderma* in the field has direct and indirect beneficial effects on commercial and food crops of interest in addition to its potential use in bioremediation and in the mycosynthesis of NPs. Few studies have been addressed the presence of metals and/or NPs in the field and their effect on *Trichoderma* and their beneficial associations, as well as on their mycoparasitic activity. Considering that an increasing number of products containing nanostructured compounds are reaching the field and consequently its eventual accumulation, they can affect the microorganisms and plants inhabiting these environments, including *Trichoderma* species. It is important to begin developing a greater number of investigations that allow us to know the specific effects whether positive or negative, that these nanostructured compounds have on the physiology, viability, and associations of *Trichoderma*. Although there will be variations depending on the nature of the nanoparticle, its shape, size, concentrations, as well as the species of *Trichoderma* and conditions tested, it is necessary to elucidate what phenotypes are at least expected in each condition to propose strategies for better use of these compounds and formulate an agrochemical that can affect different phytopathogens, but not *Trichoderma*. In this review, we focused on the effects in *Trichoderma*, but also, it is necessary to study if the presence of the NPs changes the capacity of the plant to establish specific plant-fungus interactions.

In closing, *Trichoderma* emerges as a fungus capable of biosynthesizing NPs that can be used for the formulation of agrochemicals, as well as to treat pathogens not only of plants but also of other organisms, including human pathogens. It is a fungus easy to handle and with multiple physiological and technical advantages, and the fact that is used in nanotechnology as a new line of research suggests that there are still several mechanisms to be discovered, which makes this fungus a versatile tool for future biotechnology applications.

## Author Contributions

All authors listed contributed equally and they have made a substantial, direct, and intellectual contribution to the work and approved it for publication.

## Funding

Research conducted and related to the topics of this review was supported by CONACyT grants CB 258569 and EcosNord 263456.

## Conflict of Interest

The authors declare that the research was conducted in the absence of any commercial or financial relationships that could be construed as a potential conflict of interest.

## Publisher's Note

All claims expressed in this article are solely those of the authors and do not necessarily represent those of their affiliated organizations, or those of the publisher, the editors and the reviewers. Any product that may be evaluated in this article, or claim that may be made by its manufacturer, is not guaranteed or endorsed by the publisher.
